# Inventory management performance for laboratory commodities in public hospitals of Jimma zone, Southwest Ethiopia

**DOI:** 10.1186/s40545-020-00251-1

**Published:** 2020-09-04

**Authors:** Abdi Befekadu, Waqtola Cheneke, Dereje Kebebe, Tadesse Gudeta

**Affiliations:** 1 Hospital Pharmacy, Jimma Medical Center, Jimma, Ethiopia; 2 School of Medical Laboratory Sciences, Faculty of Health Sciences, Jimma University, Jimma, Ethiopia; 3 Department of Pharmaceutics, School of Pharmacy, Faculty of Health Sciences, Jimma University, Jimma, Ethiopia; 4 Department of Social and Administrative Pharmacy, School of Pharmacy, Faculty of Health sciences, Jimma University, Jimma, Ethiopia

**Keywords:** Inventory management, Performances, Laboratory commodities, Public hospitals, Ethiopia

## Abstract

**Background:**

Maintaining an efficient and effective inventory management system ensures a reliable supply of laboratory commodities. The aim of this study was, therefore, to assess the performance of inventory management for laboratory commodities in public hospitals in the Jimma zone.

**Methods:**

A facility-based cross-sectional descriptive study, accompanied by a qualitative method, was conducted in seven public hospitals between April 30 and May 29, 2019. We collected data through document reviews (225 bin-cards), physical observation, self-administered questionnaires, and in-depth interviews. The quantitative data were analyzed using Excel spreadsheets and SPSS version 24. Fifteen key informants of different backgrounds took part in the qualitative study. The data were then analyzed using thematic analysis techniques.

**Results:**

All the public hospitals in the zone were included in the study, making a response rate of 100%. Of the total estimated bin-cards, 225 (69.9%) of them held along with the items, and only 30.4% of them filled accurately. In four of the hospitals, pharmacists determined how much to order. Five of the hospitals used average monthly consumption data to calculate purchase quantity. Over the past 6 months, four of the hospitals had placed at least one or two emergency orders. The wastage rate of the commodities in the hospitals was 27.2% and resulted in a loss of about 10,248.5 US dollars. The hospitals had met 70.6% of the criteria for proper storage conditions. Budget constraints, absence of prompt administrative support, lack of staff commitment, and frequent shortages of commodities on the part of suppliers were major bottlenecks of inventory management.

**Conclusions:**

The hospitals had weak inventory management practices, showed by inaccurate records, stock-outs (frequent emergency orders), a high wastage rate compared to national baseline statistics, and the storage conditions below the standard.

## Background

Laboratory facilities are integral parts of health care and assist the provision of quality health services through reliable diagnosis and monitoring of medical outcomes [1–3]. Nevertheless, their effectiveness relies on the sustainable availability of supplies, chemicals, and reagents with working equipment and facilities [3]. Hence, proper inventory management may help to attain the expected achievement [4].

Inventory management (IM) is a core component of supply chain management that protects healthcare against disruption of service provision [5, 6]. After commodities procured and received by the hospital organization or company, it must be delivered to the point of service provision where the customers obtain the products/services. During this process, IM informs store managers and logistic officers when to order or issue, how much to order or issue, and how to maintain adequate stock to avoid shortages and oversupplies [7–9].

IM will not seamlessly run if the management supports, which form the heart of the logistics cycle, are not operational. Information systems, human resources, supervision, monitoring, and evaluation are some of the management support measures. Information is the driver of the logistic process, including inventory control; it is ridiculous to make inventory decisions without complete and timely data [9]. Moreover, the recruitment of trained human resources, regular supportive supervision, continuous reporting, and periodic evaluation of inventory control activities help to allow the prudent use of resources and boost service levels [10, 11].

However, the quality of laboratory services in resource-limited countries such as Africa is not satisfactory [12, 13]. The issues are mostly due to a lack of resources, improper logistic activities, and inadequate administrative support [14]. For example, surveys in Rwanda, Zimbabwe, and Southern Tanzania show that health institutions have ineffective inventory control, improper storage conditions, insufficient storage capacity, and poor quality reports. As a result, they end up with erroneous order size and wrong quantity decisions, frequent stock-outs of supplies, high expiration, and increased associated costs. Additionally, a lack of skills in commodity management and limited funds for laboratory commodities become challenging [15–17].

The existing laboratory system in Ethiopia is structured within the context of the country’s general health care system. Besides health centers and health posts, this system comprises hospitals at the federal, regional, zonal, and district levels. FMoH strives to ensure that all Ethiopians have access to medical laboratory services. If there are no laboratories, a referral system should be in place for sending samples to the closest reference laboratory [18].

Despite all policy efforts, the health facilities of the country share common problems with other low-income countries. For instance, in the 2018/19 budget year, the Ethiopian Pharmaceuticals Supply Agency (EPSA) allocated 14% (24,540,777.49 USD) of pharmaceuticals budget for laboratory commodities [19]. Nonetheless, there are still shortages of laboratory supplies and reagents, including common and basic test kits [18].

Hence, as part of monitoring and evaluation, undertaking performance assessment helps to identify the weaknesses and bottlenecks of managing inventory. There are several metrics used to assess the efficiency of inventory management. The aim and context of the organization decide the choice of the indicators used [20]. Various supply chain protocols, including the Laboratory Services Assessment Tool (ATLAS), provide key performance metrics that can help for evaluating inventory management practices at both national and facility levels [21].

Previous studies used some of the indicators to assess inventory management activities for essential medicines, e.g., [10, 22–25]. However, there are few kinds of research on the management of laboratory commodity logistics yet not specific to inventory management. Thus, as far as the information of the investigators concerned, no study on the field of laboratory inventory practice has been conducted in Jimma zone. On the other hand, there are seven public hospitals in the area, one of which is a specialist hospital serving all the southwestern part of Ethiopia [26, 27]. Therefore, this study was aimed to assess inventory management performance for laboratory commodities in public hospitals in Jimma zone, Southwest Ethiopia.

## Methods

### Study area and period

The study was done in public hospitals in Jimma zone, Oromia regional state, southwestern Ethiopia. Jimma zone is one of the largest zones in the regional state of Oromia, with an area of 15,568.58 km^2^ and located 352 km away from the capital city of the country, Addis Ababa. It is divided administratively into 18 districts, including Agaro, Chora Botor, Dedo, Gera, Gomma, Guma, Kersa, Limmu Kosa, Limmu Sakka, Mana, Omo Nada, Seka Chekorsa, Setema, Shebe Senbo, Sigmo, Sokoru, Tiro Afeta, and Jimma City. The zone has 770 health institutions, including health centers (*n* = 110), health posts (*n* = 486), private clinics (*n* = 156), military hospitals (*n* = 2), private hospitals (*n* = 1), NGO clinics (*n* = 4), diagnostic laboratories (*n* = 4), and public hospitals (one referral, five district hospitals, and one zonal hospital).

### Study design

A hospital-based descriptive cross-sectional study complemented with a qualitative method was conducted from April 30 to May 29, 2019.

### Source populations, study units, and data sources

The source populations were all health facilities, laboratory commodities, health professionals, and logistics documents. The study units were public hospitals and laboratory commodities. The data sources were bin-cards, storekeepers, Health Commodity Management Information System (HCMIS)/Dagu facility, registry of expired or damaged items, and receipt vouchers (model 19).

### Inclusion and exclusion criteria

We chose public hospitals because they provide health services to larger communities and are also funded by the government directly. Besides, they demanded a larger proportion of the annual budget allocated to health facilities. In Ethiopia, supplies, kits, and reagents for advanced laboratory tests are also not accessible in lower-level public facilities, such as in health centers and health posts. Participants with a service year less than 6 months and those who were not present during the study period were excluded.

### Sampling size determination and sampling procedures

The study included all public hospitals (i.e., seven) in Jimma zone. Regarding commodities, we included the core laboratory items suggested by the Assessment Tool for Laboratory Services (ATLAS) [21], which are 46 in total. The sample size of bin-cards depended on the selected laboratory products. As a result, 46 bin-cards from each hospital were expected that could also make 322 cards. The HCMIS, model 19, and the registry of damaged/expired products were also reviewed to determine the wastage rate and value of unused items. The registration dates of the unused items were used to distinguish the corresponding model and to access the starting balance of the items and quantity received in the software (HCMIS). We also selected seven experienced storekeepers for self-administered questionnaires.

### Measurements

The key performance indicators adopted from the USAID delivery project logistic indicators assessment tool (LIAT) [28], Assessment Tool for Laboratory Services (ATLAS) [21], and integrated pharmaceuticals logistics system of Ethiopia (IPLS) [29] were used to measure the inventory management performance of the hospitals. The indicators or variables, along with their descriptions, are available in a supplementary file 1.

### Data collection procedures

Structured questionnaires and checklists developed based on the LIAT [28], ATLAS [21], and IPLS [29] were used to capture the quantitative data. Such data were obtained from store managers using self-administered questionnaires and through observation and document review using checklists. The structured questionnaire had two parts. The first part consisted of the socio-demography characteristics of the hospital pharmacy staff. And the second part contained questions dealing with facility characteristics such as stock-taking practices, ordering and receiving activities, and availability and utilization of LMIS tools. The checklists were used to collect data that could help to determine inventory accuracy, stock out rates, wastage rates, bin-card updating practices, and storage conditions of medical stores.

### Data processing and analysis

Data were entered into Epidata version 3 for cleaning and exported to SPSS version 24 for analysis. The Excel spreadsheet version 16 was also used to calculate inventory accuracy, stock out rates, and wastage rates. And then, the outputs were transferred to SPSS for further analysis. The results were then displayed using charts, graphs, and texts.

### For the qualitative study

In-depth face-to-face interviews were conducted with fifteen key informants (KIs). The number of KIs was dependent on information saturation, i.e., the interviews ended as soon as the new interviewee reiterated what had already been stated. Furthermore, the selection of the participants took into account the service year and the role in the logistic process. Accordingly, hospital storekeepers, laboratory managers, and pharmacy directors were purposively included in the interviews. One of the investigators moderated the interviews to ensure the accuracy of the details. Open-ended and probing questions were designed to explore the complexities of laboratory product management. We conducted the interviews in local languages, Afan Oromo and Amharic, and the records were audio typed. Each, on average, lasted for 45 min. Oral informed consent was received from each respondent ahead of the interview, and their confidentiality was kept private. The records were stored in separate discs protected with a password. After rehearsing, the investigators transcribed the recording into the English language. Then, one of the qualitative research experts at Jimma University verified the accuracy of the transcription. The analysis was carried out manually using a thematic technique. Codes have been assigned to variables and drawn together in a tabular format. Variables with a similar code were arranged together to formulate appropriate themes. These include problems related to logistics and information systems; issues related to product availability, administrative, and financial concerns; and supply chain staff-related problems. We then described the themes and quoted the views of the interviewees to illustrate the seriousness of the issues.

### Data quality assurance

We pretested 5% of the data collection formats at Bedelle Hospital in Bedelle Zone. The pretest was done to ensure the accuracy of the tools and to determine the approximate amount of time needed to complete the tools. Data collectors of relevant backgrounds (i.e., pharmacy and laboratory) were recruited to gather all relevant quantitative data. Moreover, the investigator provided 2 h of training for the data collectors regarding the data collection procedures by explaining the objectives of the study. The qualitative data were gathered by the investigators to maintain consistency throughout the interviews.

### Operational definitions

*Dagu facility*: is a locally developed JIS-project delivery software for recording and generating inventory reports.

*Facilities*: interchangeably used with hospitals.

*Formats*: mean inventory recording tools.

*Laboratory commodities*: include key supplies, chemicals, reagents, and kits used for running laboratory tests in the study settings.

*Laboratory products*: interchangeably used with laboratory commodities.

*Over the past 6 months*: previous 6 months as of the days of data collection.

*Over the last year*: a year just before the day of data collection, i.e., April 2018 to March 2019.

*Stocktaking practice*: physical inventory count.

*Waste*: unused laboratory commodities because of expiration, loss of shelf life, or damage.

*Working stations*: section of the medical laboratory layout in which the tests carried out.

## Results

### Characteristics of the hospitals and study participants

Six of the hospitals had received supportive supervision from the Oromia regional health bureau/Jimma zone health department in the last budget year. The area of supervision was mainly bin-card updating practices. The most recent supervision was conducted in the last month (before the study) in one of the hospitals. Concerning the participants’ profile, four of them (storekeepers) were druggists with average service years of 18.3 ± 4.6 months. All of them had received an integrated pharmaceuticals logistics system training (Table 1).

**Table 1:** The characteristics of the study participants (storekeepers) and public hospitals in Jimma zone, April 30 to May 29, 2019 (*n* = 7)

The characteristics of the hospitals		
Variables		Frequency
Received supervision from (i.e., in the last year)	FMoH^a^	1
	RHB/ZHD^b^	6
The most recent supervision	Within the last month	1
	Within the last 3 months	3
	Within the last 6 months	3
Area of supervision	Bin-card updating practices	6
	Reporting practices	4
	Storage conditions	4
	Waste management	2
The profiles of the study participants		
Qualification	Pharmacists	3
	Druggists	4
Service year	7–12 months	1
	13–18 months	3
	19–24 months	2
	Above 24 months	1
	Mean services year	18.3 ± 4.6 months
IPLS^c^ training		7

^a^Federal Ministry of Health

^b^Regional Health Bureau/Zonal Health Department

^c^Integrated pharmaceuticals logistics system

### LMIS implementation

All the hospitals implemented LMIS to maintain information about their laboratory commodities. Six of the hospitals had both a paper-based and automated system, i.e., the Dagu facility. The paper-based formats such as bin-cards, delivery notes, reports and requisition forms (RRFs), and internal facility reports and requisition forms (IFRRs) were available and used in more than five facilities (Table 2).

**Table 2:** Availability and utilization of LMIS tools per facility in selected public hospitals in Jimma zone, April 30 to May 29, 2019 (*n* = 7)

Variable			Frequency
Implemented LMIS			7
Type of LMIS implemented	Paper-based only		1
	Both paper-based and automated (HCMIS or Dagu)		6
The types of LMIS forms	Bin-cards	Available	7
		Utilized	5
	IFRRs	Available	7
		Utilized	7
	RRFs	Available	7
		Utilized	6
	Order book	Available	5
		Utilized	3
	Requisition and order voucher	Available	3
		Utilized	1
	Delivery note	Available	7
		Utilized	7

### Bin-card per items and updating practices

**Fig. 1:**
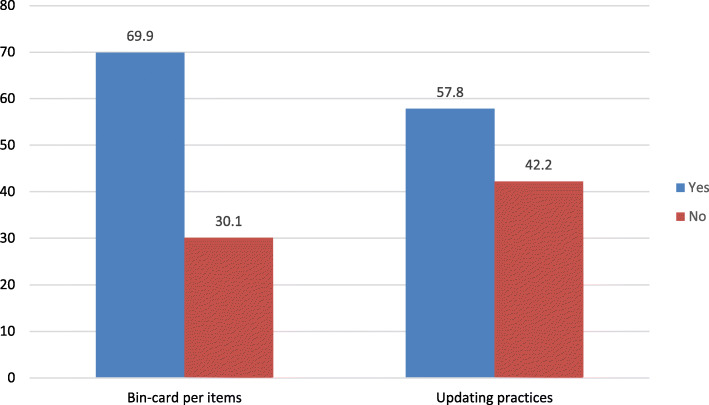
Bin-card use and updating practices in selected public hospitals in Jimma zone, April 30 to May 29, 2019

Each laboratory commodity selected for the study was physically checked for bin-cards. As a result, 225 (69.9%) of them were with bin-cards. And 130 (57.8%) of the bin-cards were updated in the last 30 days (Fig. 1).

### Physical counts of laboratory commodity and inventory accuracy rates

**Fig. 2:**
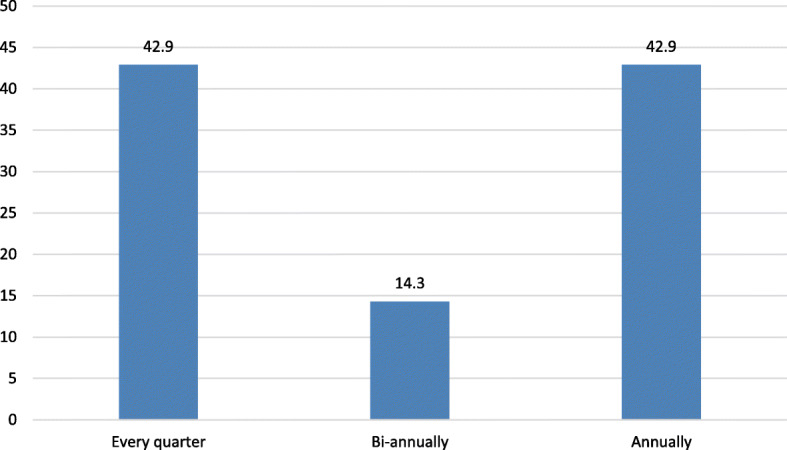
Frequency of physical counts for laboratory commodities in selected hospitals in Jimma zone, April 30 to May 29, 2019

The interview of the storekeepers indicated that six of the hospitals had done physical counts of their laboratory items in the past year, either quarterly (42.9%) or annually (42.9%) (Fig. 2).

**Fig. 3:**
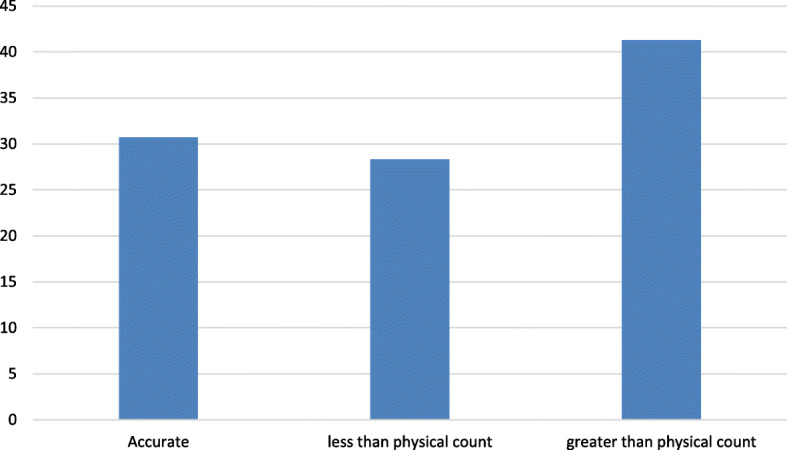
Inventory accuracy rate for laboratory commodities in selected hospitals in Jimma zone, April 30 to May 29, 2019

To determine the inventory accuracy rate, the data collectors inspected 225 bin-cards and made physical counts of the respective laboratory commodities. As a result, 93 (41.3%) bin-cards had a final balance higher than the actual numbers. Yet only 69 (30.7%) of the bin-cards had zero inconsistencies (Fig. 3).

### Ordering and receiving processes of laboratory commodities

In four of the hospitals, the quantification process is performed by pharmacists mainly using average monthly consumption (AMC) data (five hospitals). On the other hand, all the facilities had submitted their orders to the Ethiopian pharmaceuticals supply agency (EPSA), Jimma hub, and five of them had also sent to private suppliers for resupply. A purchase order in three of the hospitals initiated when the need for a product arises only. In four of the hospitals, the last report for resupply was prepared and submitted in the past 2 months before data collection. Four of the facilities had placed at least one emergency order in the last 6 months before the study (Table 3).

**Table 3:** Ordering and receiving processes of laboratory commodities in selected public hospitals in Jimma zone, April 30 to May 29, 2019 (*n* = 7)

Questions/variables		Frequency
Who determines how much to order?	Pharmacy	4
	Laboratory	1
	Both	2
Types of data elements used to calculate how much to order?	Average monthly consumption	5
	Number of tests	1
	Stock remaining in the laboratory	1
Where reports submitted to	Jimma EPSA hub	7
	Private suppliers	5
When was the last report submitted for resupply	Last month	3
	Within the past 2 months	4
Frequency of orders	Quarterly	2
	Every 6 months	2
	When need arises	3
How many emergency orders have you placed in the last 6 months?	None	3
	At least once	4
Delivery time	Less than 2 weeks	2
	2 weeks to a month	3
	Between 1 and 2 months	2

### Storage conditions

Seventeen questions/indicators were prepared to assess the storage condition of laboratory commodities. Accordingly, the hospitals met 70.6% of the storage requirements but were still below the desired criteria. Only one hospital had fulfilled 80% of the acceptable storage conditions. In five of the hospitals, cartons were either not placed on the right side or crushed due to mishandling; the items were exposed to fluorescent light and damaged. On the other side, none of the hospitals stocked flammable and dangerous goods separately from other health supplies. The storage space of the three hospitals was not adequate for the proper handling of laboratory commodities. They stored outdated or defective goods along with usable commodities. They also lacked firefighting devices. Yet all hospitals used product identity stickers, secured the store with a lock and key, and shielded products from direct sunshine at all hours of the day and during all seasons (Table 4).

**Table 4:** Storage conditions for laboratory commodities in selected hospitals in Jimma zone, April 30 to May 29, 2019 (*n* = 7)

Variables/questions	Frequency (%)
Products that are ready for distribution are arranged so that identification labels and expiry dates and/or manufacturing dates are visible.	7 (100)
Products are stored and organized in a manner accessible for first-to-expire, first-out (FEFO) counting, and general management.	5 (71.4)
Cartons and products are in good condition, not crushed due to mishandling. If cartons are open, determine if products are wet or cracked due to heat/radiation (fluorescent lights in the case of gloves, cartons right-side up).	1 (14.3)
The facility makes it a practice to separate damaged and/or expired products from usable products and removes them from inventory.	4 (57.2)
Products are protected from direct sunlight at all times of the day and during all seasons.	7 (100)
Cartons and products are protected from water and humidity during all seasons.	4 (57.2)
The storage area is visually free from harmful insects and rodents. (Check the storage area for traces of rodents [droppings or insects].)	6 (85.7)
The storage area is secured with a lock and key but is accessible during normal working hours; access is limited to authorized personnel.	7 (100)
Products are stored at the appropriate temperature during all seasons according to product temperature specifications.	6 (85.7)
The roof is always maintained in good condition to avoid sunlight and water penetration.	7 (100)
The storeroom is maintained in good condition (clean, all trash removed, sturdy shelves, organized boxes).	7 (100)
The current space and organization are sufficient for existing products and reasonable expansion (i.e., receipt of expected product deliveries for foreseeable future).	4 (57.2)
Products are stacked at least 10 cm off the floor.	5 (71.4)
Products are stacked at least 30 cm away from the walls and other stacks.	3 (42.9)
Products are stacked no more than 2.5 m high.	7 (100)
Fire safety equipment is available and accessible (any item identified as being used to promote fire safety should be considered).	4 (57.1)
Products are stored separately from insecticides and chemicals.	0
Average storage condition.	70.6%
Number of hospitals that meet the optimal storage condition, i.e., ≥ 80%.	1 (14.3)

### Availability of laboratory commodities

Based on the ending balance and physical verification, 37 (80.4%) of the commodities were available on the day of the visit. The items with zero balance include sodium chloride reagent, diethyl-ether 3%, blood agar, McConkey, Muller Hinton, powder Hb, triple sugar iron agar (TSI), oxidase reagent, and typing antisera. The retrospective review of the most recent 3 months data in bin-card and Dagu facility indicated that almost the entire laboratory items except xylene, stat-pack (HIV test kit), and examination gloves were out of stock at least for a day. The average duration of stock out was 8.5 days. Of the items, apron (plastic) and soap/hand rub alcohol were not in stores for several days, 34 and 23 days, respectively (supplementary file 2).

### Wastage rates

The registry of damaged or expired items over the last year (April 2018 to March 2019) showed that nearly half (47.8%) of the selected commodities had a history of loss or expiration. Safranin O, Gram stain reagent (iodine), Gram stain reagent (alcohol), Acid-alcohol solution, and Sensitivity antibiotic discs had high levels of waste ranging from 50 to 100%. The overall wastage rate was about 27.2%. In terms of value, around 10,248.5 US dollars lost as a result of expiration, loss of shelf life, or any other damage. Hematology autoanalyzer reagent kit (2826.9 USD), HIV test kit 1st response (2142.4 USD), and CD4 test reagent (1200.7 USD) accounted for the highest value of the loss (Table 5).

**Table 5:** Wastage rates and value of unused laboratory commodities in selected hospitals in Jimma zone, April 30 to May 29, 2019

Item description	Units	Amount wasted	*Total quantities of items in the year	Wastage rate (%)	Value of wasted items	
					Unit price (USD)	Total price (USD)
Safranin O	1 L	13	13	100	1.3	17.3
Gram stain reagent, iodine	1 L	11	14	78.6	1.3	14.6
Gram stain reagent, alcohol	1 L	23	36	63.9	2.0	45.7
Acid-alcohol solution	1 L	15	25	60	7.8	117.6
Sensitivity antibiotic discs	1 ampoule	50	100	50	7.8	388.9
Hematology autoanalyzer reagent kit	1 test	28	76	36.8	101.0	2826.9
Chemistry autoanalyzer reagent kit, GOT (AST)	1 test	23	67	34.3	26.3	603.8
HIV test kit 3rd response	1 test	17	77	22.1	37.7	640.4
HIV test kit 1st response	1 test	104	493	21.1	20.1	2142.4
Gram stain reagent, crystal violet	1 L	6	32	18.8	4.4	26.3
Carbol Fuchsin (Basic Fuchsin), 1%	1 g	11	61	18	3.9	43.0
Chemistry autoanalyzer reagent kit, glucose	1 test	10	63	15.9	18.8	187.7
Immersion oil	1 mL	18	139	13	4.9	88.6
HIV test kit 2nd response	1 test	25	216	11.6	34.0	850.8
Pregnancy test kit	1 test	31	275	11.3	13.9	429.4
Formalin, solution	1 L	15	139	10.8	2.7	40.7
Methylene blue solution	1 L	9	90	10	1.7	14.9
Methanol	Bottle	2	25	8	8.1	16.2
Chemistry autoanalyzer reagent kit, creatine	1 test	3	48	6.3	19.6	58.7
CD4 test reagents	Kit	2	35	5.7	600.4	1200.7
RPR/VDRL kit	Kit	3	150	2	13.8	41.3
Alcohol 70%	1 L	19	2352	0.8	23.8	452.9
Average wastage rate				27.2	Total (USD)	10,248.5

*Beginning balance plus total received quantities in between April 2018 and March 2019

### Qualitative results

The qualitative results were systematically analyzed and grouped into themes.

### Logistics and information systems related

The absence of standard ordering systems for different laboratory commodities made the inconsistent flow of information, which in turn caused financial decisions difficult. How much to request for some products depended on previous consumption data, the number of tests carried out, and stock out of supplies. Besides, the order pattern for these different commodities is often variable. Despite the use of the Health Commodity Management Information System (HCMIS) program, issues remain due to the reasons mentioned above. Additionally, failure of the staff in updating data in HCMIS was another bottleneck, though the facilities received continuous support from a partner, AIDS-free project.

### Product availability issues

Most of the laboratory commodities were not available at the supplier side (both EPSA and private suppliers, especially the sole suppliers), and the problems were even severe for closed system laboratory machines. These closed-circuit machines use reagents provided by a single supplier. Such sole suppliers do not deliver a sufficient quantity of reagents, mainly because of foreign exchange shortages. One of the key informants explained as follows,

*"I am a logistics officer in my hospital and worked for the past two years. Most often, the sole suppliers failed to fulfill our requirements. For example, if we request 40 units, they offer only 5% of the orders."*

As far as customer care is concerned, the interviewees stated that patients/clients often worry about having laboratory tests in a private clinic, because most of the time, the intended testing might not be carried out in public hospitals due to a lack of supplies. Since any patient coming to the hospitals may have the opportunity to visit the laboratory, the availability of tests prescribed for the patients is indeed a question of their satisfaction.

One of the laboratory managers explained it as follow

*"Most of the time, patients were complaining about lab services. Even sometimes, simple tests like stool or urine examinations may not be conducted, due to stock out of one or more reagents."*

### Administrative, financial, and facility-related problems

Hospitals have experienced extreme budget constraints in ensuring an appropriate inventory. The laboratory and pharmacy heads reported that the budget allocated to medical supplies was not adequate. Besides, the top-level management attention to pharmaceuticals, including laboratory supply management, was low. In most of the facilities, failure to timely arrange vehicles and the delayed submission of reports to suppliers were also other bottlenecks next to financial problems. The interviewees stressed that hospital managers consider laboratory items as other stationery products. One of the store managers reported as follow,

*"hospital management gives poor attention to pharmacy services. For instance, when we request a transportation service to pick commodities from suppliers, we most often have to wait at least for a month. Since the products are not timely collected, the vendors adjust the amount of supply usually to less than the required quantities. As a result, the probability of stock out at the supplier side is more likely. It, in turn, leads to stock out of items in the hospital. Hence, laboratory service providers are usually complaining about the absence of commodities to run tests."*

Concerning the service facilities, most of the refrigerators in the study hospitals were not medical refrigerators, i.e., they were home/kitchen refrigerators and did not have any embedded or external monitoring devices. No data loggers were connected to evaluate the past performance of the refrigerators. The hospitals had frequent electrical interruptions, and some of them did not have a back-up generator, or the available one was of limited capacity. Internet access was yet another problem for hospitals. One of the key informants reported

*"We requested the hospital management several times to buy a high capacity generator and to fix the internet connection issues, but no response. Since the existing generator cannot maintain the cold chain system for longer time during a power interruption, cold chain items often become useless."*

### Supply chain staffs related

The key informant revealed that in most of the hospitals, logistics/supply chain staffs, including storekeepers, were not as much committed to executing their tasks at the desired level. Most of them were negligent in maintaining logistics information. Bin-cards were not updated each time transactions conducted. Consequently, data related to the amount received, distributed/issued, damaged/expired could not be easily accessible. One of a pharmacy head described,

*"There is no obvious explanation of why store managers failed updating bin-cards; this is just due to pessimistic behavior and occasionally negligence. When requested why they did not do it, most of them replied that they are busy."*

On the other hand, some staff had lack of knowledge about laboratory commodity inventory management. The interviewees clarified that the issue had been traced to the program during formal higher education. The pharmacy curriculum had gabs in the area of laboratory commodity management. It was entirely theoretical based which could not equip with the requisite skills. There was also a challenge in hospitals in addressing skills deficits through short-term training. As a result, it was difficult for most workers to decide how much, when, and how to order laboratory products. One of a store person illuminated as follows,

*"I am a store manager in my hospital, and I have experience of more than a year in my current position. However, as far as my knowledge is concerned, there is no short-term training on the supply chain management of laboratory commodities. Hence, I always face challenges in quantifying and preparing purchase orders for these laboratory items."*

## Discussion

The ultimate goal of inventory management is to ensure an optimal supply of goods for which customers can access the services they want at a reasonable cost [8, 30]. For this reason, the provision and use of LMIS tools, the preservation of data, the regular tracking of stocks, the minimization of record errors, and the appropriate storage conditions would protect goods from stock out and wastage to accomplish the intended target [11, 31].

In the current study, almost all the required manual LMIS tools were available in the hospitals. However, not all of them were fully utilized. For example, 97 (30.1%) of the total estimated bin-cards for the selected main items were not held together with the goods. It is a large figure, particularly for high-value items where the decision to purchase impacts the use of budget. The finding is lower compared to the previous East Wollega report, where 100% of bin-cards used [10]. The reason for the disagreement between the two studies might be the difference in inventory control systems employed to manage the items. In the former study, the program drugs are managed through a forced ordering system where health facilities are expected to submit reports every 2 months to a supplier [29]. Therefore, top-level managers exclusively regulate the facilities. Nonetheless, in the current study, facilities do reporting whenever they need refilling. Hence, they may use an estimated quantity for a purchase order.

On the other hand, the use of recording tools alone could not be sufficient; they need to be updated consistently within 30 days [11, 21] and documented accurately as well. Accurate data reporting is one step in ensuring the availability of laboratory commodities. Every piece of information provides room for sound logistics decisions at each level of the supply chain [32]. Bin-cards are immediate sources of information for store managers and those interested in checking or knowing the stock status of any product. The accuracy of a bin card shows the efficiency and commitment of those involved in managing laboratory commodities [33–35]. In the present study of the total bin-cards assessed, 69 (30.7%) and 130 (57.8%) of them were completed accurately and updated in the past 30 days, respectively. These findings suggest that the hospitals did not have quality information to allow informed decision-making on inventory levels. The result for updating practices is comparable with a study done in Lesotho [36] and Addis Ababa [37], where 53% and 50% of bin-cards updated, respectively. However, the inventory record accuracy is much lower compared to the findings from a study conducted in Zimbabwe [16], wherein 60% of cards filled accurately. The in-depth interviews revealed that the store personnel in the current study were negligent and less committed to inventory management. On the other hand, the workers argued that they had a lack of skill and experience in laboratory inventory management because of the absence of training related to laboratory products.

Physical counting is one of the mechanisms for controlling inventory. It allows logisticians to verify the level of their goods, confirm if the forms are completed correctly, and to check whether unused, defective, or obsolete medicines present in the stock [38, 39]. The current study disclosed that only three hospitals conformed to the national guideline. The guideline recommends conducting a physical inventory count at least quarterly at the medical stores [3]. These are indications of loose stock control, which probably result in more wastes and stock out of supply from the facilities. Based on the qualitative findings, the potential causes for the weak practices could be the absence of proactive supervision by senior management, absence of short-term training, and lack of engagement on the part of store managers.

The other important aspect of inventory management is the preservation of appropriate product storage conditions. The storage area has to be dry, well-lit, well-ventilated, out of direct sunlight, complete with safety equipment (for example, fire extinguisher), clean, of sufficient space, and with standard pallets and shelves [40]. In the current study, six of the hospitals had met 70.6% of the required storage criteria. It is better compared to previous reports from Lesotho (57.4%) [36] and Addis Ababa (63.6%) [37] but still less than the desired threshold (i.e., ≥ 80%) [11]. Almost all the hospitals in the current study have failed in keeping dangerous chemicals and insecticides separately and shielding products like gloves from direct fluorescent light. These could be mainly because of a lack of commitment from store managers and the respective administrators.

In this study, 19.6% of the core laboratory products were not available on the day of the visit, and 47.8% of them had zero balance at least once in the past 3 months with mean stock-out duration of 8.54 days. It is suggestive of a high prevalence of out-of-stock for laboratory supplies, chemicals, and reagents. The absence of these key laboratory products just for a day has a substantial effect on service quality and customer satisfaction [38]. Besides, a single test may require multiple reagents and supplies concurrently. As a result, a lack of a specific reagent can disrupt the proposed service [41]. The findings of the present report are comparatively higher than those of the study performed among health facilities in Tanzania, where 30.8% of the goods were out of facilities [42]. The variation might be due to the different challenges the hospitals in the current study had faced. The in-depth interviews showed that the facilities experienced a lack of commitment from hospital managers, inadequate supply from sources, especially for closed-system machines, budget constraints, and transportation problems.

In the case of laboratory commodity waste, almost half of the chosen products have been damaged, expired, or lost shelf-life at least once over the last year (between April 2018 to March 2019), with a wastage rate of 27.2%. This figure is lower than a report from Rwanda, where the level of waste was estimated to be 42% [15]. Nonetheless, it is far higher than the national baseline data, i.e., less than 2% [43]. It might be due to inappropriate storage conditions, lack of facilities like standard refrigerators, and frequent power interruptions. Approximately US$ 10,248.48 lost due to the damage or expiration of laboratory products in the present study hospitals. It is of great value to the health care sector in developing countries, such as Ethiopia, where about 80% of the demand for pharmaceuticals met by imports [44]. The current finding is lower compared to the previous study conducted in public hospitals in South Africa [45], wherein US$ 44,624.98 (R 700,000) lost as a result of expiration. The variance may be due to the disparity between the types of products under investigation. The previous study had investigated the waste of medicinal products.

As a limitation, this study used data from medical stores to determine the incidence of laboratory wastes since the investigators were unable to obtain complete data from the hospital workstations.

## Conclusions

In general, the hospitals had an ineffective inventory management system for laboratory products. Many of them had inappropriate storage facilities, a high incidence of waste, stock out of core laboratory commodities, and ineffective information systems. All of them faced significant problems like budget constraints, lack of dedication from managers and staff at the operational level, absence of supportive training in laboratory logistics, insufficient infrastructure, and frequent supply interruption from vendors. These suggest weak coordination between the players in the supply chain of laboratory items. There should be continuous communications between the hospitals’ logistics officers and the respective administrators. Hence, the facilities’ managers should mobilize resources, organize training in collaboration with partners or schools, conduct regular supervision to evaluate the strength and weakness of the laboratory logistics operations, and ensure the quality of logistics information.

## Supplementary information

**Additional file 1.** The descriptions, formula, interpretation, and data sources of the main indicators/variables used in the current study.

**Additional file 2.**

## Data Availability

The data sets generated during and/or analyzed during the current study are available from the corresponding author on reasonable request.

## Abbreviations

AMC: Average monthly consumption
ATLAS: Laboratory Services Assessment Tool
EPSA: Ethiopian Pharmaceuticals Supply Agency
FMoH: Federal Ministry of Health
HCMIS: Health Commodity Management Information System
IFRR: Internal facility report and requisition forms
IPLS: Integrated pharmaceuticals logistics system
KIs: Key informants
LIAT: Logistics Indicators Assessment Tool
LMIS: Logistics Management Information Systems
RHB: Regional Health Bureau
RRF: Report and requisition forms
SPSS: Statistical Package for the Social Sciences
USAID: United States Agency for International Development
USD: United States dollar
ZHD: Zonal Health Department

## References

[1] Usaid|Deliver project. Lessons learned in managing national laboratory supply chains. 2009; Available from: http://iaphl.org/wp-content/uploads/2016/05/Lesson-Learned-Managing-National-Lab-Supply-Chains.pdf. Accessed 15 Mar 2019.

[2] Birx D, De Souza M, Nkengasong JN (2009). Laboratory challenges in the scaling up of HIV, TB , and malaria programs the interaction of health and laboratory systems , clinical research , and service delivery. AmJ Clin Pathol.

[3] Federal Ministry of Health. Federal Ministry of Health. Ethiopian hospital services transformation guidelines chapter 10 : pharmacy service Ethiopian hospital management initiative,2017; Available from: https://www.ghsupplychain.org/sites/default/files/2018-11/Pharmacy Services. Accessed 20 Mar 2019.

[4] Leung NZ, Chen A, Yadav P, Gallien J (2016). The impact of inventory management on stock-outs of essential drugs in Sub-Saharan Africa : secondary analysis of a field experiment in Zambia. PLoS One.

[5] Chopra S (2013). Supply chain management; strategy, planning and operation.

[6] Neale JJ, Tonnlin BT, Willenns SP (2004). The role of inventory in superior supply chain performance. The practice of supply chain management: where theory and application converge.

[7] Rushton A, Rushton A (2014). Handbook of logistics and distribution management.

[8] Toomey JW (2000). Inventory management: principles , concepts and techniques Materials Management I Logistics Series.

[9] USAID|Deliver project. The logistics handbook: a practical guide for the supply chain management of health commodities. USAID | Deliv Proj Task Order 1, 2011;174. Available from:http://deliver.jsi.com/dlvr_content/resources/allpubs/guidelines/LogiHand.pdf. Accessed 04 Apr 2019.

[10] Tiye K, Gudeta T (2018). Logistics management information system performance for program drugs in public health facilities of East Wollega Zone, Oromia regional state, Ethiopia. BMC Med Inform Decis Mak.

[11] Shewarega A, Dowling P, Necho W, Tewfik S, Yiegezu Y. Ethiopia: national survey of the Integrated Pharmaceutical Logistics System. 2015. p. 84. Available from: https://apps.who.int/medicinedocs/documents/s21807en/s21807en.pdf. Accessed 13 Mar 2019.

[12] Tegbaru B, Meless H, Kassu A, Tesema D, Gezahegn N, Tamene W (2004). Laboratory services in hospitals and regional laboratories in Ethiopia. Ethiop J Heal Dev.

[13] Olmsted SS, Moore M, Meili RC, Duber HC, Wasserman J, Sama P (2018). Strengthening laboratory systems in resource-limited settings. Am J Clin Pathol.

[14] Mesfin EA, Taye B, Belay G, Ashenafi A, Girma V (2017). Factors affecting quality of laboratory services in public and private health facilities in Addis Ababa, Ethiopia. EJIFCC.

[15] Lijdsman C., C. Onyango, Gatera A, Saleeb S, Tarrafeta B, Gabra M. Assessment of the health commodity supply sector in Rwanda, September 2003. Arlington; 2004. Available from: https://www.who.int/hiv/amds/en/country8.pdf. Accessed 17 Apr 2019.

[16] Nyenwa, Jabulani, Alt D, Karim A, Kufa T, Mboyane J, et al. Zimbabwe HIV & AIDS logistics system assessment. Arlington; 2005. Available from: https://www.rhsupplies.org/uploads/tx_rhscpublications/DOC95.pdf. Accessed 05 Apr 2019.

[17] Nyogea DS, Said H, Mwaigomole G, Stoeckle M, Felger I, Hatz C, et al. An assessment of the supply chain management for HIV / AIDS care and treatment in Kilombero and Ulanga districts in southern Tanzania. 2015;17(2):1–9.

[18] EHNRI. Master plan for the public health laboratory system in Ethiopia second edition (2009 – 2013). Addis Ababa,Ethiopia; 2009. Available from: http://www.ephi.gov.et/images/downloads/Ethiopia Lab Master Plan_2nd Edition.pdf. Accessed 03 May 2020.

[19] Ethiopian Pharmaceuticals Supply Agency. EPSA 2019 EFY Annual Performance Report. Addis Ababa; 2019. Available from: https://epsa.gov.et/download/plans-and-updates/?wpdmdl=2522&ind=WVBuk4ARqeGgEbqjS2eDuw-gP8nsOuECdaX3bPlsxMRtMId3V7GtsfqaTQZT36HtFqgkBFasD6MQRrmmovTuRw. Accessed 21 Mar 2019.

[20] Chae B (2009). Developing key performance indicators for supply chain: an industry perspective. Supply Chain Manag.

[21] Diallo, Abdourahmane, Teclemariam L, Felling B, Ronnow E, Hart C, et al. Assessment tool for for laboratory services. Arlington; 2017. Available from: https://www.ghsupplychain.org/sites/default/files/2019-07/ATLAS/lab/log/assess_2017.pdf. Accessed 03 Apr 2019.

[22] Getachew S. Determinants of performance of pharmaceuticals inventory control system in North wollo and Waghiimera zone. Wollo University; 2015. Available from: https://www.researchgate.net/profile/Samuel_Getachew/publication/281114501_Determinants_of_performance_of_pharmaceuticals_inventory_control_system_in_North_wollo_and_Waghiimera_zone/links/55d6d84608ae9d65948bfb49/Determinants-of-performance-of-pharmaceuticals-inventory-control-system-in-North-wollo-and-Waghiimera-zone.pdf?origin=publication_detail. Accessed 31 Jan 2019.

[23] Kefale AT, Shebo HH (2019). Availability of essential medicines and pharmaceutical inventory management practice at health centers of Adama town, Ethiopia. BMC Health Serv Res.

[24] Carasso BS, Lagarde M, Tesfaye A, Palmer N (2009). Availability of essential medicines in Ethiopia: an efficiency-equity trade-off?. Tropical Med Int Health.

[25] Fentie M, Fenta A, Moges F, Oumer H, Belay S, Sebhat Y (2014). International Journal of Research in Availability of essential medicines and inventory management practice in primary public health facilities of gondar town. Int J Res Pharmacol Pharmaceutics.

[26] Bekele G, Terefe G, Sinaga M, Belina S (2020). Utilization of non-pneumatic anti-shock garment and associated factors for postpartum hemorrhage management among health care professionals’ in public hospitals of Jimma zone, south-West Ethiopia, 2019. Reprod Health.

[27] Jimma university. JUTH background information, 2019. Available from: https://www.ju.edu.et/?q=article/specialized-hosptial. Accessed 6 Apr 2019.

[28] John Snow Inc./DELIVER. Logistics Indicators Assessment Tool (LIAT). 2008;p 1–44. Available from: https://pdf.usaid.gov/pdf_docs/Pnade735.pdf. Accessed 26 Feb 2019.

[29] Ethiopian Pharmaceuticals Supply Agency (2015). Standard operating procedures ( SOP ) manual for the integrated pharmaceuticals logistics system in health facilities of Ethiopia.

[30] Mahyadin F, Mahidin R, Asaad M, Zien R (2013). The influence of inventory management practices towards inventory management performance in Malaysian public hospitals. Int Acad Res Buss Tech.

[31] Aronovich, Dana, Tien M, Collins E, Adriano Sommerlatte LA. Measuring supply chain performance: guide to key performance indicators for public health managers. U.S. Agency for International Development. 2010. p. 62. Available from: https://www.ghsupplychain.org/sites/default/files/2019-07/Measuring%20SC%20Perf.pdf. Accessed 02 May 2019.

[32] Kagoma C, Goredema W. Assessment of Angola laboratory supply chain system; January to February 2011. Arlington; 2011. Available from: https://apps.who.int/medicinedocs/documents/s21027en/s21027en.pdf. Accessed 07 Apr 2019.

[33] Yadav P (2015). Health product supply chains in developing countries : diagnosis of the root causes of underperformance and an agenda for reform Health product supply chains in developing countries : diagnosis of the root causes of underperformance and an agenda for refo. Heal Syst Reform.

[34] Sethuraman K, Tirupati D (2005). Evidence of bullwhip effect in healthcare sector: causes, consequences and cures. Int J Serv Oper Manag.

[35] Kasimov I. Issues in logistics and supply chain management , bullwhip effect and warehouse management. 2017;p1–11. Available from: https://www.researchgate.net/profile/Ikboljon_Kasimov/publication/305143742_Issues_in_Logistics_and_Supply_Chain_Management_Bullwhip_Effect_and_Warehouse_Management/links/5b544c5fa6fdcc8dae38c321/Issues-in-Logistics-and-Supply-Chain-Management-Bullwhip-Effect-and-Warehouse-Management.pdf?origin=publication_detail Accessed 27 Mar 2019.

[36] Pharasi B (2007). Assessment of the HIV / AIDS medical supplies and laboratory commodities supply chain in Lesotho.

[37] Tilahun A, Geleta DA, Abeshu MA, Geleta B, Taye B. Assessment of integrated pharmaceutical logistic system for the management HIV/AIDS and tuberculosis laboratory diagnostic commodities in public health facilities in Addis Ababa, Ethiopia. J Pharm Care Heal Syst. 2016;3(2). 10.4172/2376-0419.1000158.

[38] Management Sciences for Health. Managing access to medicines and health technologies. Arlington: Management Sciences for Health; 2012. 1088 p. Available from: https://www.msh.org/sites/default/files/mds3-jan2014.pdf. Accessed 15 Feb 2019.

[39] John Snow Inc. The supply chain manager’s handbook:a practical guide to the management of health commodities. Arlington; 2017. Available from: http://supplychainhandbook.jsi.com/wpcontent/uploads/2017/02/JSI_Supply_Chain_Manager%27s_Handbook_Final-1.pdf. Accessed 23 Feb 2019.

[40] Ethiopian Food and Drug Authority. Guidelines for good storage practices. Ethiopia; 2015 p. 71. Available from: https://apps.who.int/medicinedocs/documents/s23059en/s23059en.pdf. Accessed 09 Mar 2019.

[41] Usaid | Deliver Project. Quantification of health commodities: laboratory commodities companion guideforecasting consumption of laboratory commodities. Arlington; 2011. Available from: https://www.psmtoolbox.org/wp-content/uploads/2017/11/QuanHealCommLabo.pdf. Accessed 04 Apr 2019.

[42] Kagaruki GB, Kamugisha ML, Kilale AM, Kamugisha E (2018). Supply chain management of laboratory supportive services and its potential implications on the quality of HIV diagnostic services in Tanzania. Tanzan J Health Res.

[43] Federal Ministry of Health. National pharmacy service , pharmaceuticals supply chain and medical device management monitoring and evaluation framework. Addis Ababa; 2019. Available from: http://www.moh.gov.et/ejcc/sites/default/files/2019-07/National Pharmacy Service%2C Pharmaceuticals Supply Cain and Media Equipment Managment Monitering and Evaluation Framework.pdf. Accessed 25 Mar 2019.

[44] Ministry of Health, Ministry of Industry. National strategy and plan of action for pharmaceutical manufacturing development in Ethiopia ( 2015 – 2025 ) Developing the pharmaceutical industry and improving access. Addis Ababa; 2015. Available from: https://www.who.int/phi/publications/Ethiopia_strategy_local_poduction.pdf. Accessed 25 Mar 2019.

[45] Kachwee M, Hartmann D. Hospital supply chain management and optimization. 2013. Available from: https://pdfs.semanticscholar.org/5824/638f1c8b6aa41adead95ca62772b20cecc4a.pdf. Accessed 22 Apr 2019.

