# Supplementary material for: Inventory management performance for laboratory commodities in public hospitals of Jimma zone, Southwest Ethiopia
Source: J Pharm Policy Pract. 2020 Sep 4;13:49. doi: 10.1186/s40545-020-00251-1 (PMC7472577; doi:10.1186/s40545-020-00251-1)
Supplement: Supplementary file 1 — Additional file 1. The descriptions, formula, interpretation, and data sources of the main indicators/variables used in the current study. [file 40545_2020_251_MOESM1_ESM.docx]

Supplementary file 1. The descriptions, formula, interpretation, and data sources of the main indicators/variables used in the current study

| ***Indicators/variables*** | ***Descriptions*** | ***Formula*** | ***Interpretation*** | ***Data sources*** |
| --- | --- | --- | --- | --- |
| LMIS implementation | Helps to measure whether facilities preserve inventory details | % age availability and utilization of inventory control formats/tools.  The tools include IFRR*, RRF**, HCMIS, Models (19 & 22) delivery notes, etc. | an information system is well implemented if the tools are 100% available and utilized | Storekeepers,  Observation |
| Record update practice | Registering a change in stock status as products are issued or received, and physical counts are carried out. | $=\frac{total number of items of which bin-card updated}{total number of items that have bin card}$**100* | Bin-cards have to be updated in the last 30 days to be deemed up to date | Bin-cards |
| Physical  inventory | The hospitals' physical counting of items. Helps to identify stock on hand, expired or damaged goods, fast or slow-moving items | the frequency or percentage of inventory physical counts | Better inventory control is the more frequent stock count | Storekeepers |
| Stockout  rate | It helps to measure the level of product availability in the specified time | $\frac{No. of items that experienced stock out in a specific period}{total number of products expected to be availed}$*100 | availability>80%  means high | Bin-cards, HCMIS, Observation |
| Wastage  rate; | It helps to assess the level of product damage, expiration or loss of shelf life | $=\frac{Unusable physical stock}{beginning balance plus total received quantities}$ *100 | Wastage rate greater than 2% means there is high damage/expiration in the hospitals | Registry of unused products, model 19,& HCMIS |
| Value of wasted items | It helps to determine the amount of dollars lost as a result of damage or expiration of the commodities | The multiplication of the unit price of the commodities by the total amount of the same items.  The final total value is obtained by adding the values of all the commodities | Although no losses are expected, certain losses can occur and the ratio of these losses to the overall value gained can suggest shortcomings in storage or product policies | Registry of damaged or expired items, Model 19, |
| Ordering and receiving process | It helps to know how the facilities process orders like the frequency of orders, where to order, how to order | Percentage or proportion of the variables | The facilities have good inventory practice if for instance reports submitted timely, reduced emergency orders and the like | Storekeepers |
| Storage  conditions; | Aids to measure the performance of facilities for the proper storing of goods | $\frac{number of storage criteria fulfilled}{a total number of storage criteria}$*100 | >80% positive response to the criteria =>good storage condition | Storekeepers,  physical observation |

**Sources**: adapted from LIAT (2008), ATLAS (2017), and IPLS (2015)

| **Note**: We used 17 storage criteria to measure store conditions,  **Abbreviations:**  IFRR-internal facility report and requisition form,  RRF-report and requisition form  HCMIS-health commodity management information system |
| --- |
