# Supplementary material for: Inventory management performance for laboratory commodities in public hospitals of Jimma zone, Southwest Ethiopia
Source: J Pharm Policy Pract. 2020 Sep 4;13:49. doi: 10.1186/s40545-020-00251-1 (PMC7472577; doi:10.1186/s40545-020-00251-1)
Supplement: Supplementary file 2 — Additional file 2. [file 40545_2020_251_MOESM2_ESM.docx]

| List of key laboratory products | Units | Days out of stock in the past 3 months | Availability on the day of visit  Yes/No |
| --- | --- | --- | --- |
| Field stain A reagent | 1 gram | 7 | Yes |
| Field stain B reagent | 1 gram | 10 | Yes |
| Carbol Fuchsin (Basic Fuchsin), 0.03% | 1 gram | 14 | Yes |
| Carbol Fuchsin (Basic Fuchsin), 1% | 1 gram | 4 | Yes |
| Sodium chloride reagent | 1 gram | 7 | No |
| Formalin, solution | 1 liter | 8 | Yes |
| Diethyl-ether 3% | 1 liter | 12 | No |
| India ink | 1 mL | 15 | Yes |
| Potassium hydroxide, reagent | 1 gram | 5 | Yes |
| Pregnancy test kit | 1 test | 12 | Yes |
| HIV test kit Stat-pack | 1 test | 0 | Yes |
| HIV test kit Abon | 1 test | 2 | Yes |
| HIV test kit Bio Line | 1 kit | 1 | Yes |
| CD4 test reagents | 1 kit | 5 | Yes |
| RPR/VDRL kit | 1 test | 19 | Yes |
| Hepatitis screening kit | 1 test | 19 | Yes |
| Chemistry autoanalyser reagent kit, glucose | 1 test | 11 | Yes |
| Chemistry autoanalyser reagent kit, creatine | 1 test | 11 | Yes |
| Chemistry autoanalyser reagent kit, GOT (AST) | 1 test | 10 | Yes |
| Hematology autoanalyser reagent kit | 1 test | 7 | Yes |
| Gram stain reagent, crystal violet | 1 liter | 1 | Yes |
| Gram stain reagent, iodine | 1 liter | 1 | Yes |
| Gram stain reagent, alcohol | 1 liter | 1 | Yes |
| Safranin O | 1 liter | 9 | Yes |
| Methylene blue solution | 1 liter | 7 | Yes |
| Acid-alcohol solution | 1 liter | 1 | Yes |
| Blood agar | 1 bottle | 8 | No |
| McConkey | 1 bottle | 8 | No |
| Muller Hinton | 1 bottle | 8 | No |
| Powder Hb | 1 bottle | 8 | No |
| TSI (triple sugar iron agar) | 1 bottle | 8 | No |
| Oxidase reagents | 1 gram | 8 | No |
| Typing antisera | 1 mL | 8 | No |
| Sensitivity antibiotic discs | 1 ampoule | 1 | Yes |
| Methanol | 1 liter | 7 | Yes |
| Xylene | 1 liter | 0 | Yes |
| Immersion oil | 1 mL | 2 | Yes |
| Alcohol 70% | 1 liter | 6 | Yes |
| Hand soap/Hand rub alcohol | 1 bar of soap | 23 | Yes |
| Safety box | 1 box | 9 | Yes |
| Examination gloves | 1 pair | 0 | Yes |
| Waste container | 1 receptacle | 13 | Yes |
| Eye goggles | 1 pair of goggles | 12 | Yes |
| Face Mask | 1 mask | 7 | Yes |
| Apron (plastic) | 1 apron | 34 | Yes |
| Laboratory coats | 1 coat | 21 | Yes |
| Average stock out and duration | | $\frac{393days}{46 items}$  =8.5 days | $\frac{9 items}{46 items}*100=19.6\%$ |
